# Incidence and Consequences of Near-Drowning–Related Pneumonia—A Descriptive Series from Martinique, French West Indies

**DOI:** 10.3390/ijerph14111402

**Published:** 2017-11-17

**Authors:** Laura Cerland, Bruno Mégarbane, Hatem Kallel, Yanick Brouste, Hossein Mehdaoui, Dabor Resiere

**Affiliations:** 1 Department of Emergency Medicine, University Hospital of Martinique, Fort-de-France, 97261 Martinique, France; lauracerland@gmail.com (L.C.); Yannick-Jean.BROUSTE@chu-martinique.fr (Y.B.); 2 Department of Medical and Toxicological Critical Care, Lariboisière Hospital, Paris-Diderot University, INSERM UMR-S 1144, 75013 Paris, France; bruno.megarbane@aphp.fr; 3 Intensive Care Unit, Hospital Centre of the French Guiana, 97306 Cayenne, France; Hatem.kallel@ch-cayenne.fr; 4 Intensive Care Unit, University Hospital of Martinique, Fort-de-France, 97261 Martinique, France; hossein.mehdaoui@chu-martinique.fr

**Keywords:** drowning, pneumonia, aspiration, predictive factor, fatality

## Abstract

Drowning represents one major cause of accidental death. Near-drowning patients are exposed to aspiration that may result in pneumonia with life-threatening consequences. We designed this descriptive study to investigate the frequency, nature, and consequences of post-drowning pneumonia. One hundred and forty-four near-drowning patients (33 children and 111 adults) admitted during four years to the University Hospital of Martinique, French Indies, were included. Patients presented pre-hospital cardiac arrest (41%) and exhibited acute respiratory failure (54%), cardiovascular failure (27%), and lactic acidosis (75%) on admission. Empirical antibiotics, as decided by the physicians in charge, were administered in 85 patients (59%). Post-drowning early onset bacterial pneumonia was diagnosed as “possible” in 13 patients (9%) and “confirmed” in 22 patients (15%). Tracheal aspiration revealed the presence of polymorphous pharyngeal flora (59%) or one predominant bacteria species (41%) including *Enterobacter aerogenes*, *Enterobacter cloacae*, *Staphylococcus aureus*, *Pseudomonas aeruginosa*, *Aeromonas hydrophilia*, and *Morganella morgani*. Despite adequate supportive care, drowning resulted in 45 fatalities (31%). Early onset bacterial aspiration pneumonia (either possible or confirmed) did not significantly influence the risk of death. In conclusion, near-drowning–related bacterial aspiration pneumonia seems rare and does not influence the mortality rate. There is still a need for practice standardization to improve diagnosis of post-drowning pneumonia and near-drowning patient management.

## 1. Introduction

Drowning is a major public health issue with an annual incidence of 500,000 victims and 360,000 fatalities worldwide [1,2]. In France, including metropolitan France and overseas departments and territories, drowning currently causes 1800 deaths every year. It is one of the leading causes of accidental mortality in children and adolescents. 

Drowning is defined as acute respiratory failure resulting from immersion or submersion of the airways in a liquid medium, usually water, and leading to death [3]. Near-drowning results in multiple complications, including aspiration pneumonia, that often lead to life-threatening conditions. Prognosis remains poor in the absence of rapid and appropriate management, especially in the event of pre-hospital cardiac arrest. 

About 90% of near-drowning patients aspirate the aquatic environment. Thus, only a minority may avoid aspiration and possibly be at lower risk of pneumonia [4]. In various acute pathologies involving aspiration as in drowning, bacterial pneumonia does not develop consistently following aspiration [5,6]. In addition, given the potential severity of aspiration and the absence of reliable clinical or laboratory markers separating non-infectious pneumonitis from bacterial aspiration pneumonia, patients routinely receive antibiotics without prior bacterial sampling to diagnose infection if suspected [7].

Post-drowning pneumonia is poorly understood and documented. Its exact incidence is unknown, while it probably causes significant morbidity contributing to the high rate of fatality attributed to drowning. The objectives of this study were to describe the frequency, nature, and consequences of near-drowning-related pneumonia. 

## 2. Methods

We conducted a retrospective descriptive study including all near-drowning patients admitted to the emergency and intensive care unit (ICU) of the University Hospital of Fort-de-France, Martinique, in the French West Indies over a period of four years (2012–2015). We included near-drowning patients regardless of their age and clinical severity; however, we excluded drowning patients taken to hospital who died on the spot. Patients were selected from among those encoded with a final diagnosis of “drowning” using the International Statistical Classification of Diseases and Related Health Problems, ICD-10. Data were collected from the selected patients’ records. 

Severity of drowning was classified according to the four-stage classification of Van Berkel et al. [8], adopted by the French Institute of Health Watch (InVS). This classification was based on baseline clinical features obtained on admission from history, physical examination, arterial blood gas analysis, and chest radiograph. Class I or aqua stress corresponded to immersion without inhalation when baseline examination was negative. Class II corresponded to mild immersion with hypoxia, i.e., with baseline examination positive but no need for mechanical ventilation on admission. Class III corresponded to severe immersion with consciousness disorder, acute respiratory distress, and major hypoxemia requiring mechanical ventilation on admission. Class IV corresponded to patients suffering from cardiopulmonary arrest.

Post-drowning bacterial pneumonia was considered in patients displaying the following during the first 48 h after hospital admission: (1) fever, chills, coughing, or muco-purulent secretions; (2) infiltrates on the chest X-ray persisting on day 2; (3) hyperleucocytosis and growing inflammatory biological syndrome; and (4) positive pulmonary sample or blood culture (*a fortiori* to bacteria known to give post-drowning pneumonia). Our criteria were in agreement with the classification proposed by Ender et al. [4]. Bacterial pneumonia was considered as possible if conditions 1–3 were met and as confirmed if conditions 1–4 were met. Definitions of organ failure and modalities of patient management followed the internationally recognized criteria and guidelines.

Descriptive data are presented as percentages for qualitative variables and median (25–75 percentiles) for quantitative variables. We determined the predictive factors of death using univariate analysis. Quantitative data were compared using Mann-Whitney tests and qualitative data using Chi-2 tests. All tests were performed using Prism version 6.0 (GraphPad Software, San Diego, CA, USA). *p*-values < 0.05 were considered as significant.

## 3. Results

One hundred and forty-four patients (102 males/42 females; 33 children/111 adults; median age: 45 years (19–60)) were included in this study over four years. Drowning occurred in seawater (N = 120; 83%), swimming pool (N = 15, 10%), fresh (non-salty) pond water (N = 8, 6%), or dirty swampy water (N = 1, 1%). Seventy-eight patients (54%) presented known past morbidities including hypertension (N = 20, 14%), epilepsy (N = 19, 13%), diabetes (N = 16, 11%), heart disease (N = 8, 6%), and obesity (N = 8, 6%). The causes of drowning included accidental fall (N = 36, 25%), malaise of cardiac origin (N = 31, 22%), alcohol abuse (N = 11, 8%), seizure onset (N = 10, 7%), accidents during nautical sporting activities (N = 8, 6%), trauma (N = 3, 2%), and suicide attempt (N = 3, 2%). The exact reason for drowning remained unknown in 42 cases (29%).

Sixty-one patients (41%) presented pre-hospital cardiac arrest and were in deep coma (Glasgow coma score: 3/15) on hospital admission (Table 1). Near-drowning patients presented acute respiratory failure (N = 77, 54%), cardiovascular failure (N = 37, 27%), and lactic acidosis (N = 108, 75%). Acute respiratory distress syndrome (ARDS) developed in 23 patients (16%). Ninety-two patients were mechanically ventilated using invasive (N = 64, 44%) or non-invasive ventilation (N = 28, 19%). Two patients required veno-venous extracorporeal membrane oxygenation for refractory hypoxemia. Based on the evaluation of the severity of drowning on admission, patients were classified as class I (N = 33, 23%), class II (N = 19, 13%), class III (N = 31, 22%), and class IV (N = 61, 42%). 

Antibiotics were initiated empirically on hospital admission in 85 patients (59%) and included amoxicillin/clavulanic acid (92%), cefotaxime (6%) and cefotaxime + metronidazole (2%). Post-drowning early-onset bacterial pneumonia was actually diagnosed as “possible” in 13 patients (9%) and “confirmed” in 22 patients (15%). Pulmonary sampling (i.e., tracheal aspiration) revealed the presence of polymorphous pharyngeal flora in the majority of these 22 cases (59%). One predominant bacteria species was isolated in only nine patients (41%). Identified bacteria were *Enterobacter aerogenes* (N = 2), *Enterobacter cloacae* (N = 2), *Staphylococcus aureus* (N = 2), *Morganella morgani* (N = 1), *Pseudomonas aeruginosa* (N = 1) and *Aeromonas hydrophilia* (N = 1). No significant relationship was noted between whether or not antibiotics were initiated and the class of drowning.

**Figure 1:**
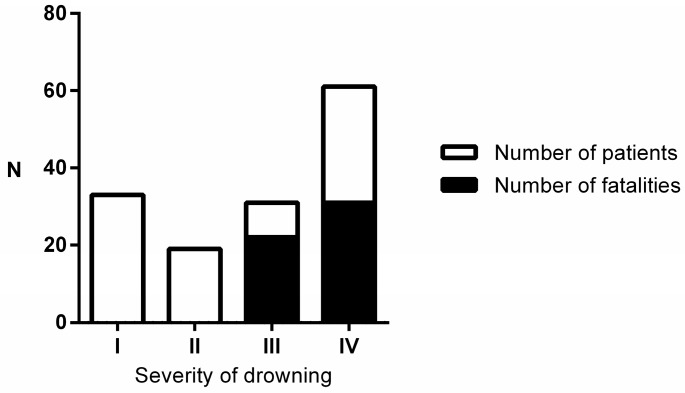
Distribution of fatalities in relation to the severity of drowning (N = 144).

Forty-five patients (31%) died (Table 2). The distribution of fatalities in relation to the severity of drowning is represented on Figure 1. The length of hospital stay was seven days (2–18). The factors on admission associated with increased risk of hospital death were a more elevated age (*p* = 0.0003), the onset of pre-hospital cardiac arrest (*p* < 0.0001), Glasgow coma score of three (*p* = 0.009), ARDS (*p* = 0.01), and lactic acidosis (*p* = 0.05). Early onset bacterial aspiration pneumonia (either possible or confirmed) did not significantly influence the risk of death.

## 4. Discussion

As shown in our series, drowning is responsible for life-threatening complications leading to a high fatality rate, especially in patients with circulatory arrest on admission. We showed that near drowning–related bacterial pneumonia is not so frequent and does not influence the outcome.

The majority of drownings in Martinique take place at sea and in swimming pools. Usually, reasons for drowning depend on the age of the victim [1,2]. In children aged less than 13 years of age, the lack of supervision and not knowing how to swim are the main causes. With advancing age, as in our series, the proportion of medical causes of drowning increases including malaise, seizures and heart problems. However, accidental fall, trauma, and sporting activities represent major drowning causes in adults.

Given the pathophysiology of drowning [4], pulmonary infection in near-drowning patients should not be a rare event. However, the exact proportion of near-drowning victims who develop pulmonary infection has never been clearly evaluated and varies according to the series. Consistent with our series, Oakes et al. reported 16 pulmonary infections among 40 near-drowning patients (40%) [9], while Lee et al. found only 12 cases of pulmonary infections among their 102 near-drowning patients (11%) [10]. Another study analyzed the occurrence of pulmonary infection among children who suffered inhalation, including 13 admitted for near-drowning [11]: seven of these 13 patients (54%) developed pneumonia, but the exact bacteria responsible for the pulmonary infection were not specified. In our series, we found a rather low rate of near drowning–related bacterial pneumonia (24%), with confirmed diagnosis in only 15% of the patients. Interestingly, the majority of drownings in our patients occurred in seawater while the literature suggests that pulmonary infections are less frequent after drowning in salt water than in swampy or contaminated water [8]. Additionally, bacterial infections may have been underestimated due to the almost systematic administration of prophylactic antibiotics before pulmonary sampling in the ICU (82% of our cases). However, we took into account the evolution of the inflammatory parameters over the next 48 h to confirm or invalidate possible bacterial pneumonia diagnosis according to the criteria we used [4].

Diagnosis of bacterial pneumonia following aspiration is rather difficult. As in all medical conditions possibly complicated by aspiration, history, physical examination, and laboratory and radiographic findings cannot reliably predict bacterial pneumonia or distinguish it from chemical pneumonitis [12]. Interestingly, a recent clinical investigation showed that only half the comatose patients receiving mechanical ventilation with suspected bacterial aspiration pneumonia had this diagnosis based on telescopic plugged catheter sampling [6]. The authors demonstrated that, in the absence of clinical, laboratory, or radiologic evidence of bacterial aspiration, pneumonia patients did not require antibiotics, and that in patients with suspected bacterial aspiration pneumonia, stopping empirical antibiotic therapy when routine telescopic plugged catheter sampling recovered no microorganisms was nearly always effective. In this study, this strategy was not used to diagnose post-drowning pneumonia.

Onset of pulmonary infection in the near-drowning patient represents a potentially serious event [4]. However, the exact resulting lethality remains controversial. In our study, nine patients presented a documented pulmonary infection; three of these patients died (33%). Death rate was not significantly influenced by the onset of bacterial pneumonia. One possible explanation that may have lowered the influence of post-drowning pneumonia on mortality is that the severity of drowning by itself was important as evidenced by the elevated percentages of stage III and IV patients with cardiac arrest and cardiovascular failure.

In this study, the decision to administer prophylactic empirical antibiotics, left to the physicians in charge, was routinely taken. However, the usefulness of prophylactic antibiotics is still controversial [4]. Studies that investigated this issue are old, hard to access, retrospective, and involved limited data sets. In a retrospective study including 105 near-drowning patients, Van Berkel et al. showed that prophylactic antibiotics were not protective [8]. In contrast, in another series from Singapore, none of the 17 ICU patients who received prophylactic antibiotics developed pneumonia [10]. Thus, although not systematically recommended, empirical antibiotics should be considered using a low threshold, aiming to treat the probable pathogens. If indicated, the choice of empiric antibiotic therapy is also not obvious. Antibiotics should cover both endogenous pathogens (oropharynx, upper-airways, and digestive tract in case of vomiting) and pathogens found in the aquatic environment that differ according to the type of water involved (salt, fresh or contaminated) [4]. To date, there is no study evaluating the usefulness of prophylaxis using broad-spectrum antibiotics.

Our study presents significant limitations mainly due to its retrospective methodology. We only performed an univariate analysis including the available parameters with no missing information. We cannot rule out the fact that other non-collected clinical parameters may have influenced the patient’s death; however, missing values did not allow us to include them in a multivariate analysis. Additionally, as explained above, administration of empiric antibiotics on admission before pulmonary sampling in the ICU may have altered the microbiological results. However, one strength in this study is its real-life design, including a relatively large number of near-drowning adult and children patients.

## 5. Conclusions

Drowning is responsible for an elevated fatality rate despite adequate supportive care in the ICU. Near drowning–related bacterial aspiration pneumonia seems rare and does not influence the mortality rate. Due to controversial data in the literature, standardization is still awaited in the practices of prevention, diagnosis and management of pneumonia resulting from near-drowning. There is a clear need for comprehensive prospective studies to develop common, harmonized, and population-friendly protocols for near-drowning patient management.

## Figures and Tables

**Table 1:** Major features and complications presented by the near-drowning patients (N = 144).

Clinical Features	Patients (N = 144)
Pre-hospital cardiac arrest	61 (41%)
Lactic acidosis	108 (75%)
Consciousness impairment	84 (58%)
Acute respiratory failure	77 (54%)
Cardiovascular failure	37 (27%)
Early onset bacterial pneumonia	35 (24%)
Acute respiratory distress syndrome	23 (16%)
Death	45 (31%)

**Table 2:** Characteristics of the near-drowning patients according to their final outcome (N = 144).

Clinical Parameters	Survivors (N = 99)	Non-Survivors (N = 45)	*p*
Age	39 years (6–56) ^1^	56 years (40–66)	0.0003
Gender (F/M)	31%/68%	16%/84%	NS ^2^
Classes of drowning severity (I, II, III, IV)	33%/19%/31%/17%	100%	<0.0001
Pre-hospital cardiac arrest	15%	100%	<0.0001
Glasgow Coma Score on admission	6 (3–12)	3 (3–4)	0.009
Acute respiratory distress syndrome on admission	5%	40%	0.01
Lactic acidosis on admission	64%	100%	0.05
Early onset bacterial aspiration pneumonia	25%	22%	NS

^1^ Data presented as median (percentiles 25–75); ^2^ NS, not significant.
